# Application of imaging studies for orbital tumors: a practical review and representative cases

**DOI:** 10.3389/fopht.2026.1822331

**Published:** 2026-04-30

**Authors:** César Fernández, J. Matthew Debnam, Bita Esmaeli

**Affiliations:** 1 Oculoplastics Clinic, Department of Ophthalmology, Hospital Universitario Universidad Autónoma de Nuevo León, Monterrey, Mexico; 2 Department of Neuroradiology, The University of Texas M.D. Anderson Cancer Center, Houston, TX, United States; 3 Oncologic Ophthalmic Plastic and Orbital Surgery, Houston, TX, United States; 4 Department of Clinical Sciences, University of Houston Tilman J. Fertitta Family College of Medicine, Houston, TX, United States

**Keywords:** lacrimal gland tumors, ocular adnexal lymphoma, orbital lymphoma, orbital rhabdomyosarcoma, orbital tumors, pleomorphic adenoma of lacrimal gland, secondary tumors of orbit

## Abstract

Orbital imaging has undergone a significant transformation over the past few decades, evolving from basic radiographic techniques to sophisticated cross-sectional imaging modalities. Historically, conventional radiography was limited to visualizing only the bony structures of the orbit or radiodense foreign bodies, making it inadequate for detecting soft tissue pathology. Advances in imaging over the last several decades—first with computed tomography (CT) in the 1970s and later with magnetic resonance imaging (MRI) in the 1980s—have revolutionized orbital diagnostics by enabling detailed visualization of both osseous and soft tissue structures. Magnetic resonance imaging (MRI) has emerged as the preferred modality for evaluating orbital tumors and other soft tissue lesions. Its superior soft tissue resolution, multiplanar imaging capability, and ability to characterize lesion morphology and extent make it crucial in clinical practice. MRI is especially valuable in differentiating benign from malignant lesions, evaluating involvement of critical structures such as the globe, optic nerve, extraocular muscles, and vascular components, and assessing disease at the orbital apex or with suspected intracranial extension. Advanced MRI techniques, including diffusion-weighted imaging (DWI) and dynamic contrast-enhanced (DCE) MRI, further enhance diagnostic accuracy. In pediatric populations, MRI holds particular importance due to its lack of ionizing radiation and its effectiveness. In contrast, while CT offers superior resolution of bony anatomy and is useful for detecting calcifications and fractures, it is somewhat limited in soft tissue differentiation and involves exposure to ionizing radiation. While CT examination can be tailored to evaluate bone or soft tissues, orbital CT is typically reserved for cases requiring detailed osseous assessment, acute trauma settings, or for patients who cannot undergo an MRI. MRI creates high-resolution images by detecting signals emitted by hydrogen nuclei when exposed to a strong magnetic field and radiofrequency pulses. Differences in signal based on tissue composition and water content are captured and converted into high-resolution images. Interpreting orbital lesions requires standardized terminology.

## Background

Orbital imaging has undergone a significant transformation over the past few decades, first with computed tomography (CT) in the 1970s and later with magnetic resonance imaging (MRI) in the 1980s. This has enabled detailed visualization of both osseous and soft tissue structures (1).

**Figure 1:**
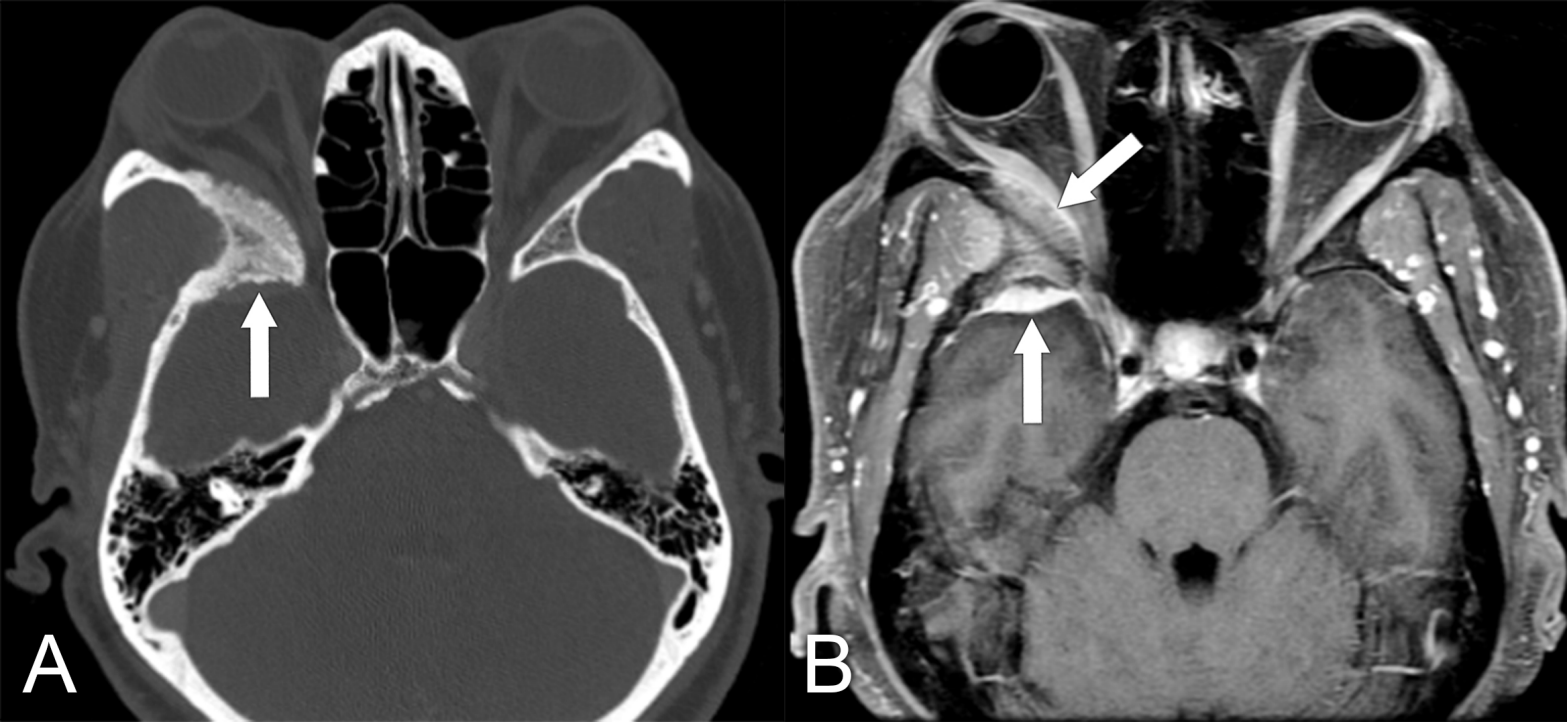
Imaging findings in a patient who presented with slowly progressive proptosis of the right eye. **(A)** Axial CT, bone window, shows hyperostosis of the lateral right orbital wall and sphenoid bone (arrow). **(B)** Axial T1 postcontrast MRI with fat suppression shows an enhancing mass involving the right sphenoid bone, posterior orbit, and middle cranial fossa (arrows). The diagnosis was confirmed to be sphenoid wing meningioma.

MRI is the preferred imaging method for evaluating orbital tumors and soft tissue lesions due to its excellent soft tissue detail, capability to image in multiple planes, and effectiveness in assessing lesion characteristics and extent. It aids in differentiating benign from malignant lesions and assessing involvement of critical structures such as the optic nerve and orbital apex, with advanced techniques further improving accuracy, such as diffusion-weighted imaging (DWI), apparent diffusion coefficient (ADC), and dynamic contrast-enhanced (DCE) MRI (2). MRI may be preferred in children with orbital and ocular tumors because it avoids radiation exposure; however, it often requires sedation in pediatric patients. It is also contraindicated in patients with metallic foreign bodies, and it may not be tolerated in patients with claustrophobia. It is also more expensive and takes longer to perform compared to CT. Computed tomography is the preferred modality for assessing bony lesions and/or bony involvement by tumors, but for most orbital tumors, MRI provides more detailed information regarding the soft tissue extension of the mass (**Figures 1A, B**). CT is also used in acute orbital trauma settings (3, 4).

Although MRI may be considered the gold standard for assessment of orbital tumors in the developed world, complementary imaging modalities may play an important role in specific scenarios and in environments where access to MRI may not be readily available. Ultrasound is useful for evaluation of superficial and anterior orbital lesions, such as dermoid cysts and vascular lesions, allowing differentiation of solid vs. cystic lesions and assessment of internal vascularity with Doppler imaging (5). CT is valuable for fibro-osseous lesions, lesions with osseous involvement, and tumors with calcifications, such as retinoblastoma (6). Angiography, including MRI angiography, is reserved for vascular lesions, such as arteriovenous malformations and carotid-cavernous fistulae (6). Finally, positron emission tomography-CT (PET-CT) is useful for systemic staging to detect metastatic disease in solid tumors and for orbital lymphoma staging, and importantly, for assessment of response to treatment in patients who already have metastatic disease (7).

MRI creates high-resolution images by detecting signals emitted by hydrogen nuclei when exposed to a strong magnetic field and radiofrequency pulses. Differences in signal based on tissue composition and water content are captured and converted into high-resolution images. Interpreting orbital lesions requires standardized terminology: hyperintense (brighter), hypointense (darker), or isointense (similar) relative to surrounding tissues, such as the extraocular muscles. T1-weighted images provide excellent anatomical detail, especially for fat, which appears hyperintense. T2-weighted images are sensitive to fluid content and are useful for fluid-rich lesions such as cysts, inflammatory lesions, or vascular lesions, which often appear hyperintense. Fat-suppressed sequences enhance lesion visibility by eliminating the high signal from fat, thereby aiding detection of enhancing lesions and improving contrast resolution. Contrast-enhanced T1- and T2-weighted imaging, particularly with fat suppression, is essential for assessing vascularity and internal lesion characteristics. Additionally, DWI and ADC maps help evaluate tissue cellularity and diffusion restriction; for example, lymphomas typically show restricted diffusion due to high cellularity, appearing hyperintense on DWI and hypointense on ADC maps. Together, these MRI techniques allow for accurate differentiation of orbital pathologies and help to narrow the differential diagnosis (4).

## Diagnostic features of various orbital pathologies

Orbital tumors represent a wide and complex spectrum of conditions, including benign and malignant neoplasms, as well as inflammatory, vascular, and infectious disorders (**Table 1**). Due to overlapping clinical presentations, imaging studies—particularly MRI—play a critical role in establishing a differential diagnosis (8). In a large series by Shields et al., which analyzed 1,264 patients referred for suspected orbital tumors, vasculogenic lesions were the most common (17%), followed by lymphoid lesions, lacrimal gland lesions, optic nerve lesions, meningeal lesions, metastatic lesions, peripheral nerve lesions, and melanoma (9). Among adults, the most frequent orbital tumors include lymphoproliferative lesions, cavernous venous malformations, and metastatic lesions. Inflammatory conditions such as thyroid eye disease and idiopathic orbital inflammation are also commonly seen and may mimic neoplastic processes. In pediatric populations, typical lesions include dermoid cysts, rhabdomyosarcoma, and retinoblastoma with orbital extension (9).

**Table 1:** Common orbital pathologies: clinical presentation and MRI features.

Pathology	Clinical presentation	Key MRI features
Lymphoma	Painless, slowly progressive proptosis; often in elderly patients	T1: Isointense to muscle T2: Mild to moderate hyperintense Postcontrast: Homogeneous enhancement DWI: Restricted diffusion; well-defined, noninfiltrative margins
Cavernous venous malformation (CVM)	Painless, gradual proptosis; middle-aged adults	T1: Isointense T2: Markedly hyperintense Postcontrast: Progressive, heterogeneous enhancement; well-circumscribed intraconal mass
Idiopathic orbital inflammation (IOI)	Acute painful proptosis, eyelid edema, diplopia	T1: Isointense T2: Variable, often mildly hyperintense Postcontrast: Diffuse enhancement involving muscles and tendons; orbital fat stranding; may show restricted diffusion
Thyroid eye disease (TED)	Bilateral proptosis, lid retraction, diplopia; associated with Graves’ disease	T1: Isointense T2: Hyperintense in active phase Postcontrast: Muscle belly enhancement with tendon sparing; symmetric EOM enlargement
Lacrimal gland tumors	Superolateral mass, globe displacement; may be painful if malignant	Benign: Well-defined, homogeneous enhancement (e.g., pleomorphic adenoma) Malignant: Irregular margins, heterogeneous enhancement, infiltration, or perineural spread
Optic nerve sheath meningioma (ONSM)	Gradual vision loss, optic atrophy, optociliary shunt vessels	T1: Isointense T2: Variable Postcontrast: “Tram-track sign” with peripheral sheath enhancement; sparing of optic nerve core
Dermoid cyst (pediatric)	Congenital, painless, palpable mass near orbital rim or sutures	T1: Hyperintense due to fat T2: Variable No enhancement Well-circumscribed, noninfiltrative; often superotemporal location
Rhabdomyosarcoma (pediatric)	Rapid-onset proptosis, eyelid swelling, ophthalmoplegia	T1: Isointense T2: Hypo- to hyperintense Postcontrast: Heterogeneous enhancement; aggressive, poorly defined margins; may show bony erosion

While CT imaging is useful for identifying the presence of a mass and is often used as an initial imaging study due to its speed, relatively low cost, and availability, MRI offers superior soft tissue contrast resolution, can more confidently differentiate between various lesions, and is critical in narrowing the differential diagnosis. A thorough clinical history, in conjunction with high-resolution MRI, remains essential for accurate diagnosis and treatment planning. In the following paragraphs, basic information about each of the common orbital lesions is provided, along with representative imaging studies.

## Lymphoproliferative disorders

**Figure 2:**
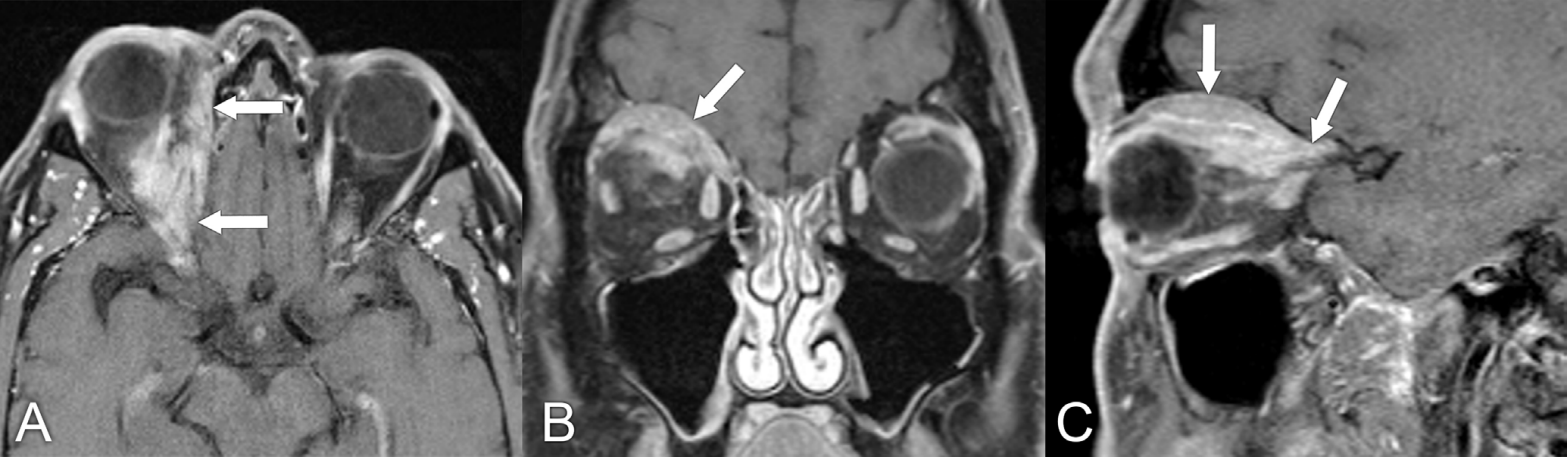
Magnetic resonance imaging in a 61-year-old man with slowly progressive right eye proptosis for the past year. **(A–C)** Axial, coronal, and sagittal, T1 postcontrast MRIs with fat suppression reveal a large infiltrating right superior orbital mass extending posteriorly to involve the orbital apex and superior orbital fissure, with further extension toward the right cavernous sinus (arrows). An anterior orbitotomy and biopsy of the lesion confirmed the diagnosis of marginal zone lymphoma. Systemic workup was otherwise negative for lymphoma.

**Figure 3:**
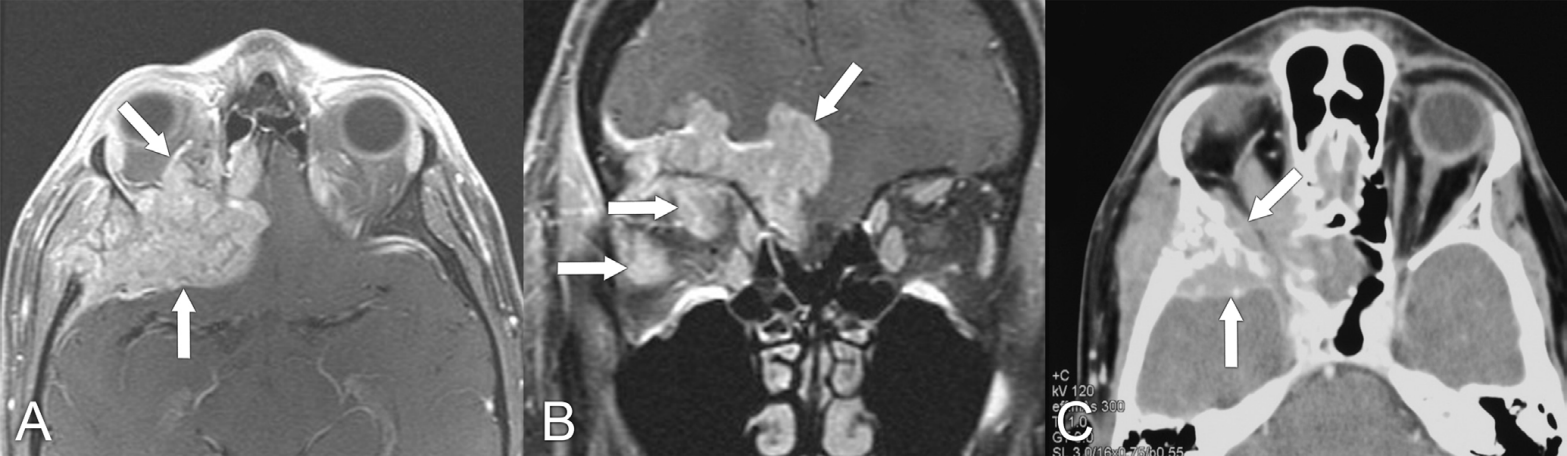
A 37-year-old man presented with right facial swelling, progressive proptosis of the right eye that developed in the last 2–3 months prior to presentation. The patient had restriction of upward gaze and complained of a pressure sensation in the right periorbital area. **(A, B)** Axial and coronal T1 postcontrast MRIs with fat suppression show an infiltrative mass with bony destruction of the right orbital apex and roof, with involvement of the extraocular muscles and extension into the frontal lobe (arrows). **(C)** CT with contrast, soft tissue window, in the same patient again shows the mass in the right orbit and middle cranial fossa with underlying bone destruction (arrows). The differential diagnosis included a metastatic lesion to the orbital apex vs. a primary bony cancer such as osteosarcoma. A biopsy was done through a craniotomy and confirmed the diagnosis of diffuse large B-cell lymphoma. This case represents an atypical presentation of lymphoma.

**Figure 4:**
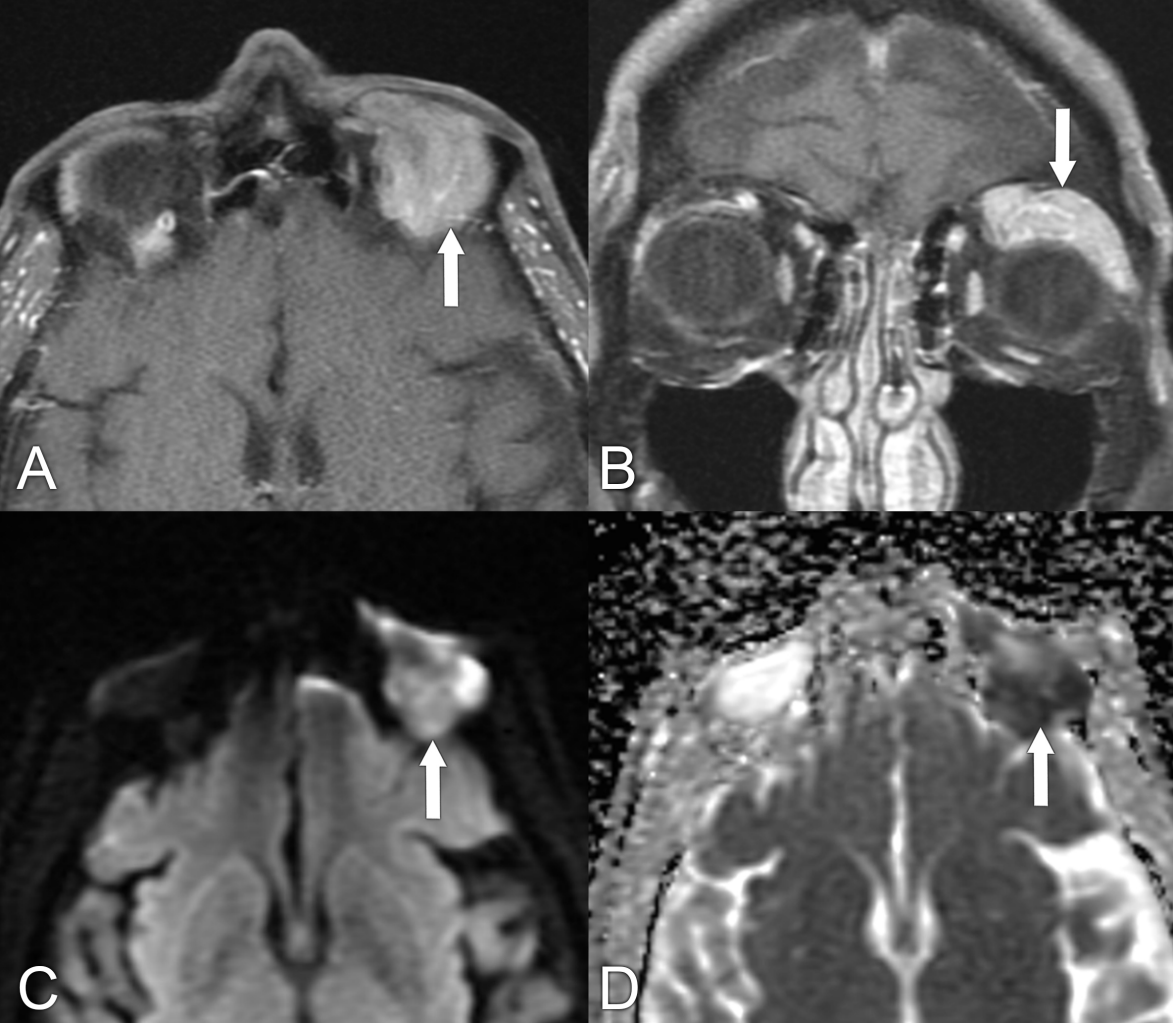
A 76-year-old man with left conjunctival mucosa-associated lymphoid tissue (MALT) lymphoma presented with ptosis. **(A, B)** Axial and coronal T1-weighted postcontrast MRIs with fat suppression show a homogeneously enhancing mass in the superior left orbit that molds around the globe (arrows). **(C, D)** Axial diffusion-weighted and apparent diffusion coefficient MRIs show restricted diffusion within the mass, with signal hyperintensity and hypointensity, respectively (arrows).

Orbital lymphomas are among the most common malignant tumors of the orbit in adults, particularly in older individuals (10). Clinically, they typically present with a slowly progressive, unilateral, painless proptosis and are often located in the anterior orbit or the superior-lateral quadrant. They can involve the lacrimal gland in 40% of cases, sometimes displacing the globe (11). Patients with orbital lymphoma may experience diplopia, eyelid swelling, or droopiness. Orbital lymphoma usually molds to adjacent orbital structures without bony destruction (12, 13). On MRI, these lesions are usually isointense to muscle on T1-weighted sequences and hyperintense on T2-weighted images, with homogeneous, intense enhancement after gadolinium administration (**Figures 2A–C**). Chronic lesions can show osseous remodeling and expansion of the bony walls of the orbit. Bony destruction is rare but can occur in atypical cases and in more aggressive histological variants of lymphoma (**Figures 3A–C**). DWI and ADC can aid in differentiating orbital inflammatory lesions from lymphoma. Reflecting high tissue cellularity, lymphomas demonstrate marked diffusion restriction with high signal on DWI and corresponding low ADC with values ranging from 0.6 to 0.8 × 10^−3^ mm^2^/s (**Figure 4**). In contrast, inflammatory lesions show less restriction due to edema and loose cellularity, resulting in higher ADC values, generally around 1.0 to 1.3 × 10^−3^ mm^2^/s. Several studies have proposed diagnostic thresholds with ADC values below 0.75–0.80 × 10^−3^ mm^2^/s for lymphoma and values above 0.9–1.0 × 10^−3^ mm^2^/s supportive of inflammatory etiology, with diagnostic accuracies approaching 90%–95% (14–17).

## Pleomorphic adenoma of the lacrimal gland

**Figure 5:**
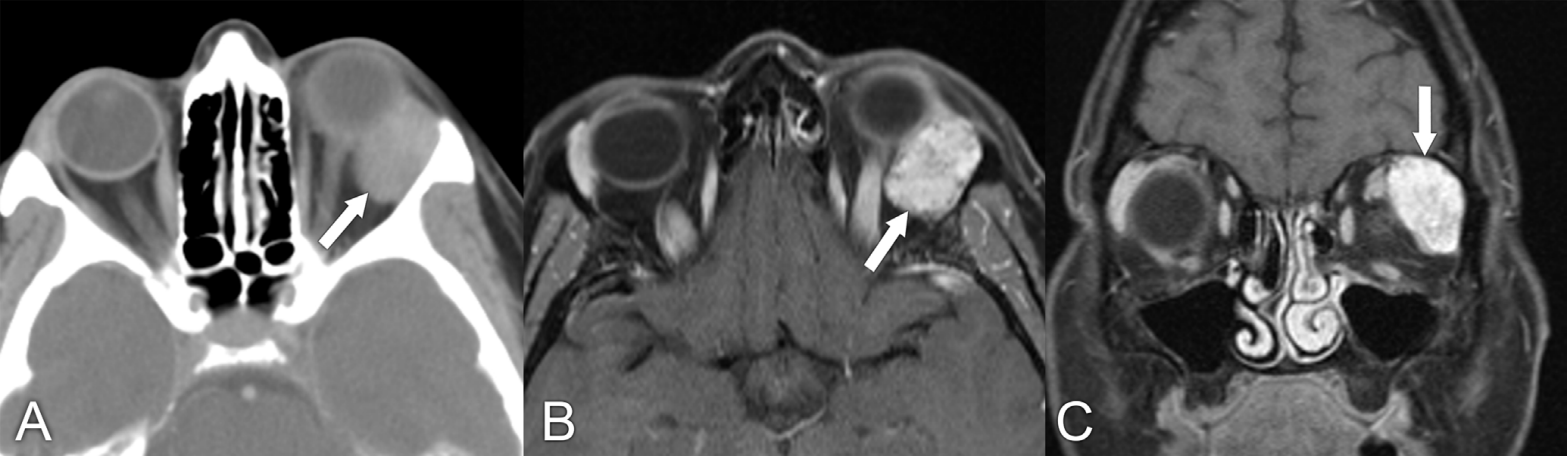
A 45-year-old woman with about a 1-year history of left upper eyelid droopiness and proptosis. **(A)** CT with contrast, soft tissue window, shows a 2.5 cm × 2.5 cm × 1.5 cm homogeneously enhancing ovoid mass of the left lacrimal gland with no bony destruction (arrow). **(B, C)** Axial and coronal T1 postcontrast MRIs with fat suppression show a well-circumscribed heterogeneously enhancing lesion of the left lacrimal gland (arrows). The diagnosis was confirmed to be pleomorphic adenoma of the lacrimal gland based on evaluation of the surgical specimen.

**Figure 6:**
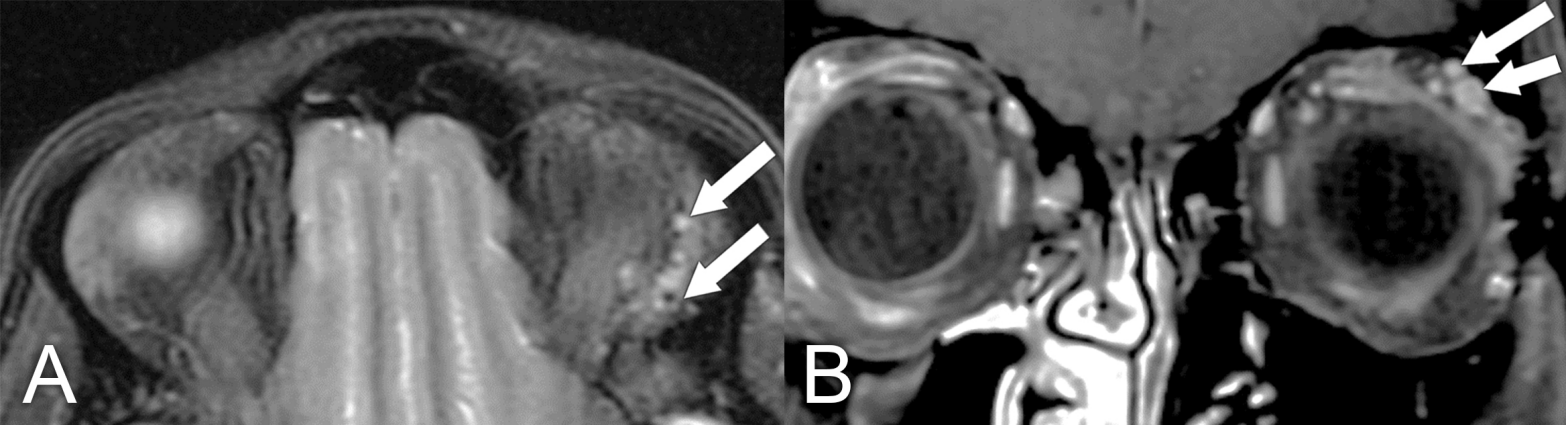
MRI in a 29-year-old woman who had undergone resection of a pleomorphic adenoma. More than 5 years later, she presented with some proptosis and multifocal recurrence in the surgical bed. **(A)** Axial T2 MRI with fat suppression shows multiple T2 hyperintense nodules in the superotemporal left orbit (arrows). **(B)** Coronal T1 postcontrast MRI with fat suppression in the same patient shows several enhancing nodules consistent with multifocal recurrent pleomorphic adenoma (arrows). The patient had surgical debulking of this multifocal lesion. The nodules were confirmed to be benign recurrent pleomorphic adenoma. It was not possible to remove all of these nodules, given their diffuse and extensive involvement of orbital soft tissue.

Lacrimal gland tumors, such as pleomorphic adenoma or adenoid cystic carcinoma, present as superolateral orbital masses causing downward and medial globe displacement. Pleomorphic adenoma (PA), also known as benign mixed tumor, is the most common benign epithelial neoplasm of the lacrimal gland, typically originating from its orbital lobe. It primarily affects middle-aged adults between 40 and 50 years of age, with about two-thirds of cases occurring in women (18). Clinically, PA presents as a slow-growing, painless mass in the superolateral orbit, often persisting for more than 12 months and causing slow-onset proptosis and downward displacement of the eye (19). Remodeling of the bony lacrimal fossa may be present on CT but should not be mistaken for an indicator of malignancy (**Figure 5A**) (20–22). On MRI, a typical PA appears as a well-circumscribed lesion with heterogeneous signal intensity on T2-weighted images and heterogeneous contrast enhancement (**Figure 5B, C**). Importantly, PAs do not show restricted diffusion on DWI, which helps distinguish them from malignant lesions. Although benign, pleomorphic adenomas carry a risk of recurrence and malignant transformation (carcinoma ex pleomorphic adenoma), particularly when only a biopsy or incomplete excision is performed (**Figures 6A, B**). Therefore, complete surgical removal and thorough histopathological evaluation, especially for capsular invasion, are critical.

## Lacrimal gland adenoid cystic carcinoma

Lacrimal gland adenoid cystic carcinoma (LGACC) is the most common malignant epithelial tumor of the lacrimal gland, accounting for approximately 29% of all epithelial tumors and up to 50% of malignant lacrimal gland neoplasms, yet it is a very rare cancer (23). It typically presents in the fourth decade of life with a firm, often painful mass in the superolateral orbit. The presence of pain is frequently attributed to perineural invasion, a hallmark of LGACC, but is not uniformly present in all cases (24). Histologically, adenoid cystic carcinoma (ACC) can exhibit tubular, cribriform (“Swiss cheese” pattern), basaloid, or comedo-like architectures. Survival rates vary significantly across reports, with improved survival reported in recent decades with multidisciplinary management, including eye-sparing surgery followed by radiation therapy in selected patients (25). In a recent report of 52 patients with LGACC, the 5- and 10-year disease-specific survival for patients who had eye-sparing surgery was 87% and 76%, respectively, and was higher than the disease-specific survival rates for patients who underwent orbital exenteration (62.3% and 57.5%, respectively). A significant difference was also noted in DSS between basaloid (solid) vs. nonbasaloid histologic subtypes, with worse survival in basaloid histology (24).

**Figure 7:**
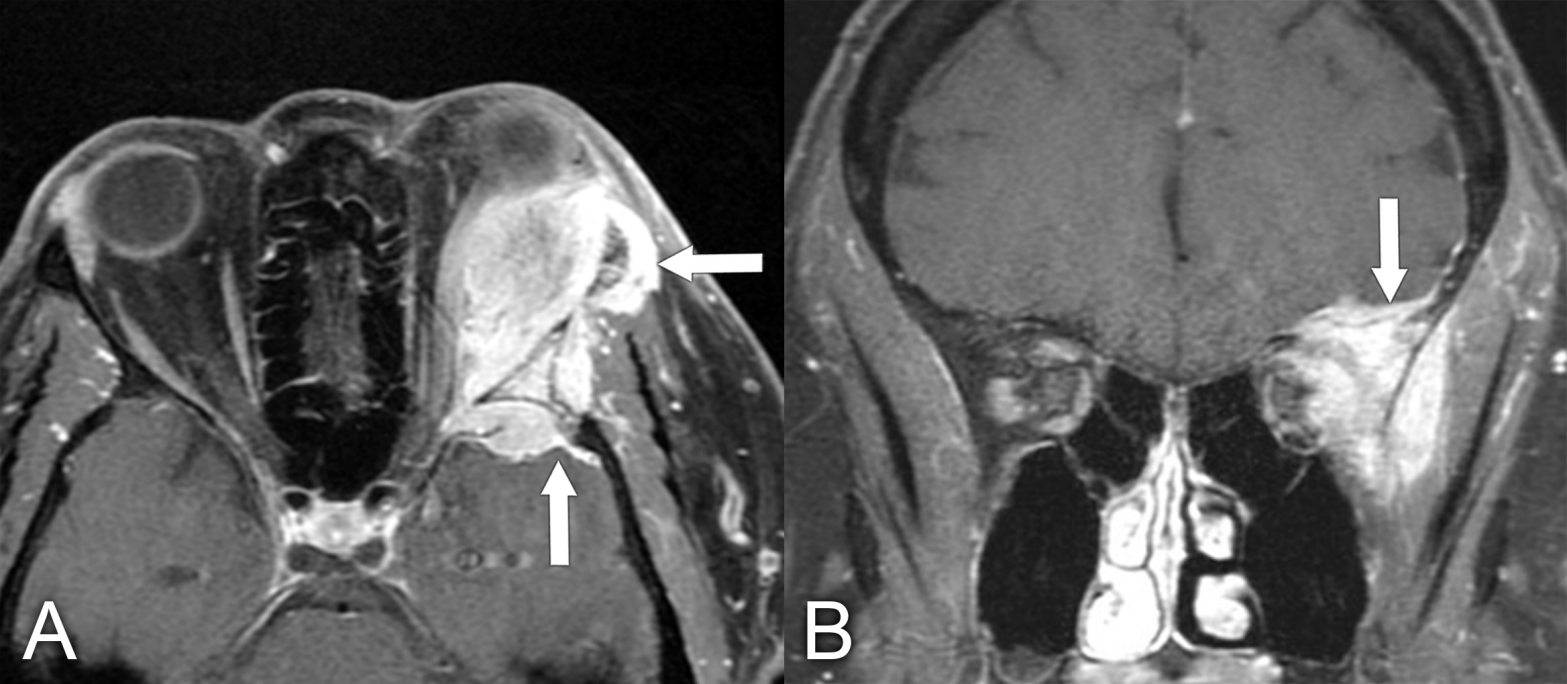
MRI findings in a 59-year-old man with locally advanced (T4) adenoid cystic carcinoma of the lacrimal gland. **(A)** Axial T1 postcontrast MRI with fat saturation shows lateral extension of the mass into the temporal fossa and posterior extension into the middle cranial fossa (arrows). **(B)** Coronal T1 postcontrast MRI with fat saturation shows extension of the lesion into the anterior cranial fossa (arrow).

**Figure 8:**
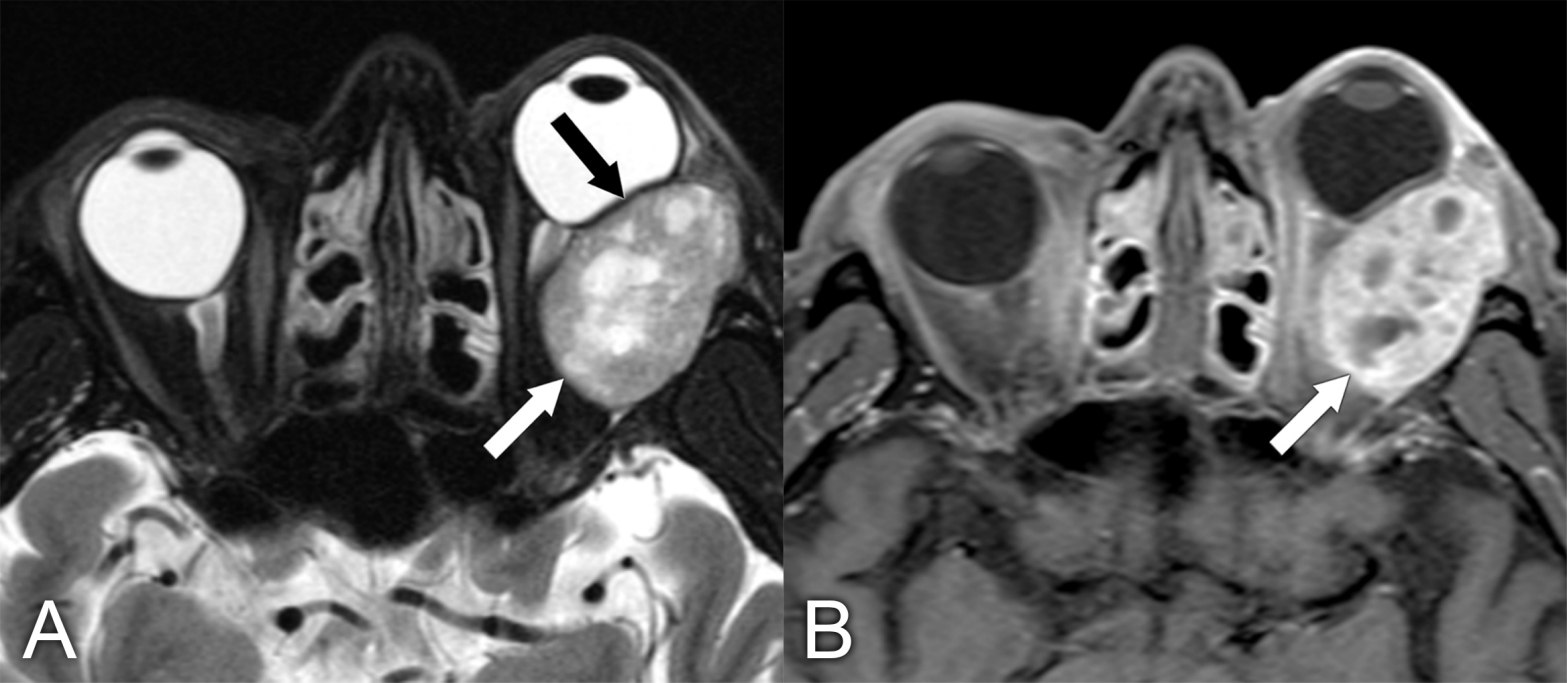
A 55-year-old man presented with a 2-year history of progressive proptosis and downward displacement of the left eye and some blurry vision. Imaging characteristics were not typical for lacrimal gland adenoid cystic carcinoma. **(A)** Axial T2 MRI with fat saturation shows a heterogeneous lesion (white arrow) causing mass effect upon the globe (black arrow) with proptosis. **(B)** Axial T1 postcontrast MRI with fat saturation shows heterogeneous enhancement of the mass (arrow). The final diagnosis, based on pathologic assessment of the surgical specimen, was adenoid cystic carcinoma.

**Figure 9:**
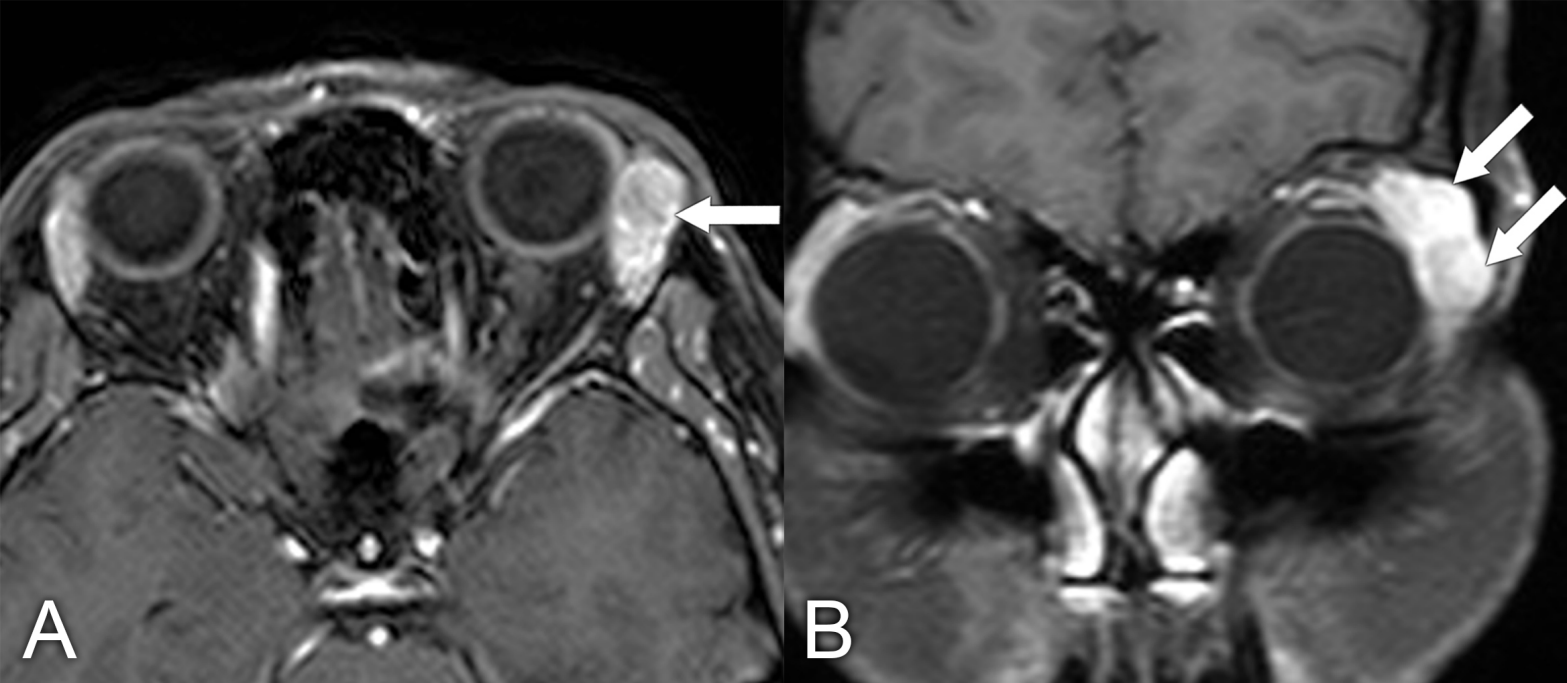
MRI findings in a 55-year-old woman who presented with early-stage (T2) lacrimal gland adenoid cystic carcinoma. **(A)** Axial T1 postcontrast MRI with fat suppression shows an elongated lesion with posterior extension that enhances with contrast (arrow). **(B)** Coronal T1 postcontrast MRI with fat suppression shows that the lesion is multilobular and abuts the superior rectus and lateral rectus muscles (arrows).

Imaging features are often nonspecific on CT, but bony destruction can be seen in association with lacrimal gland adenoid cystic carcinoma. MRI is the preferred imaging modality as it can help delineate the soft tissue extension of the lesion. This is particularly important in recent decades, as eye-sparing surgery can be offered to selected patients with LGACC (13, 14). On MRI, ACC typically appears hypointense on T1-weighted images, variable (hypo- to hyperintense) on T2-weighted sequences, and typically demonstrates prominent contrast enhancement. **Figure 7** shows MRI findings in a patient with early-stage lacrimal gland ACC. [eighth edition American Joint Committee on Cancer (AJCC) T2], whereas **Figure 8** shows MRI findings in a patient with advanced-stage disease (eighth edition AJCC T4) (**Figures 7A, B**, **9A, B**). Some patients present with atypical features such as rounded, well-circumscribed lesions (**Figures 8A, B**). DWI can aid in differentiation; ACC shows low ADC values (mean ~ 0.8 × 10^−^³ mm²/s), which are lower than those seen in benign tumors such as pleomorphic adenoma but higher than those seen in lymphomas (~ 0.6 × 10^-^³ mm²/s) (20, 21, 26).

## Cavernous venous malformation

**Figure 10:**
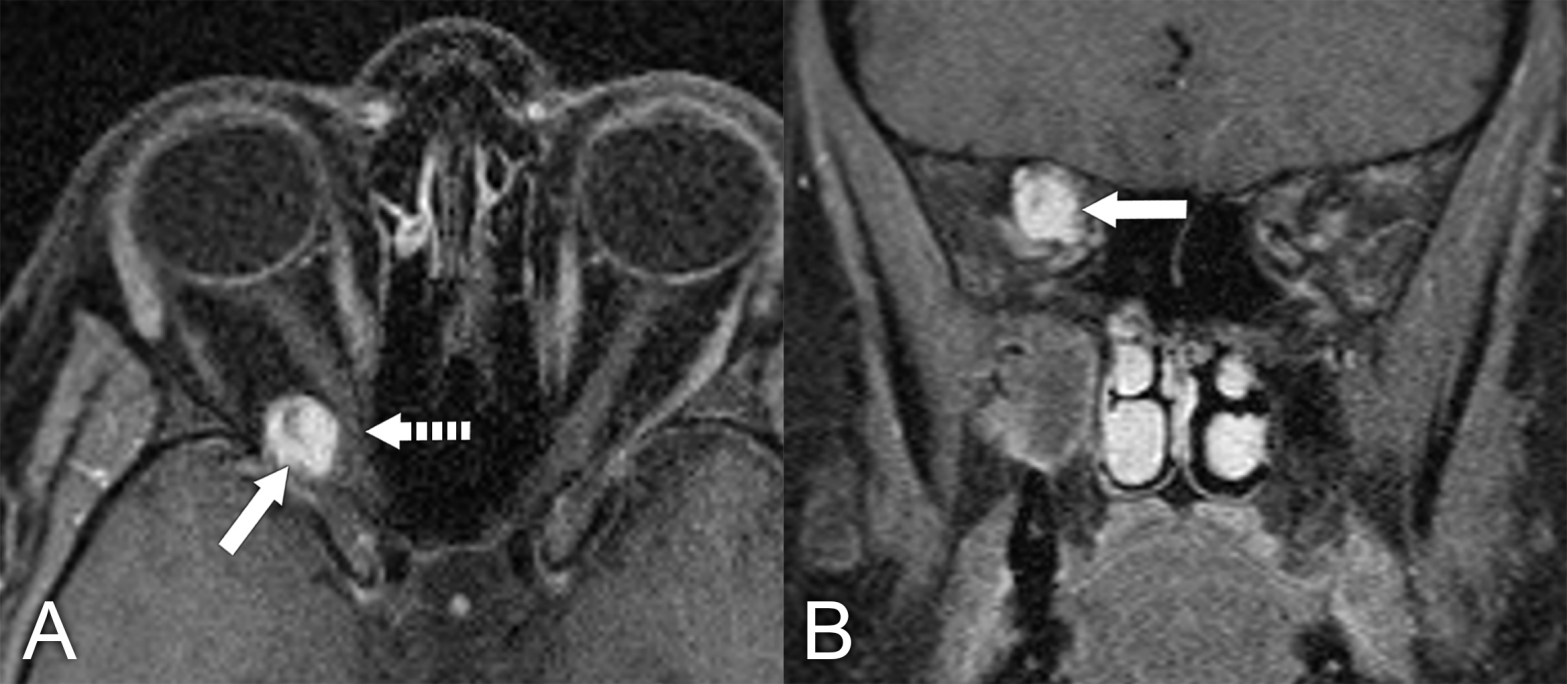
Magnetic resonance imaging findings in a 47-year-old woman who presented with a gradual onset of blurry vision in the right eye. **(A)** Axial T1 postcontrast MRI with fat saturation shows a heterogeneously enhancing mass in the right orbital apex measuring 1.3 cm × 1.1 cm × 0.9 cm (solid arrow) with compression of the optic nerve (dashed arrow). **(B)** Delayed coronal T1 postcontrast MRI with fat saturation shows gradual filling of contrast in the lesion (arrow). The presumed diagnosis of cavernous venous malformation was made based on the characteristic radiologic findings. The patient was treated with stereotactic radiotherapy, and her visual function improved in response to this treatment.

Cavernous venous malformation (CVF), formerly known as cavernous hemangioma, represents the most common benign orbital tumor in adults. Cavernous venous malformation typically affects middle-aged women and presents as painless, slowly progressive proptosis (27). The lesion is characteristically intraconal in location, most often between the optic nerve and lateral rectus muscle. CVF can grow with hormonal changes during pregnancy and adolescence (28). MRI demonstrates a well-circumscribed encapsulated mass that is T1-isointense and markedly T2-hyperintense. After contrast administration, the lesion demonstrates gradual, progressive enhancement on postcontrast sequences over time due to slow venous blood flow within the lesion (**Figure 10**) (29–31).

## Idiopathic orbital inflammation

**Figure 11:**
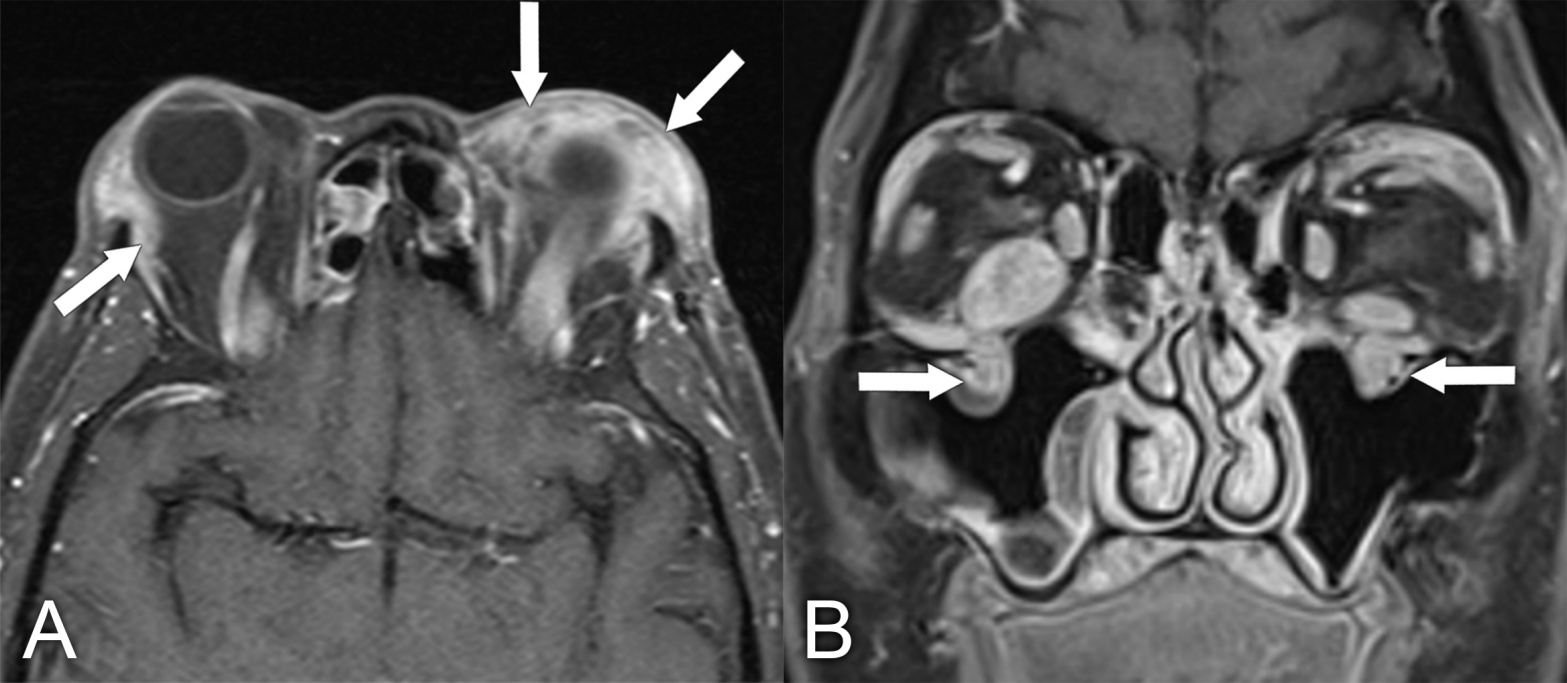
MRI findings in a 61-year-old man who presented with a several-year history of periorbital edema and proptosis. A biopsy of the orbital lesion confirmed a diagnosis of sclerosing orbital inflammatory syndrome, with more than 40% of plasmacytes positive for IgG4 and elevated serum IgG4 levels, making the diagnosis of IgG4-related disease very likely. **(A)** Axial T1 postcontrast MRI with fat saturation shows diffuse soft tissue inflammation in both orbits (arrows). **(B)** Coronal T1 postcontrast with fat saturation shows enlargement of the infraorbital nerves bilaterally, which is quite characteristic of IgG4-related disease (arrows).

**Figure 12:**
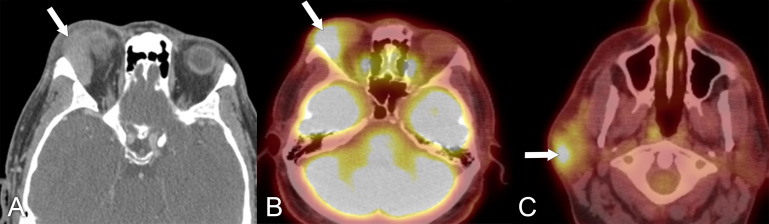
Imaging findings in a 49-year-old male patient who presented with right-sided proptosis and some orbital pain. **(A)** Axial CT with contrast, soft tissue window, shows right lacrimal gland enlargement (arrow). A biopsy of this lesion was performed, confirming the diagnosis of sclerosing orbital inflammatory syndrome, IgG4-related disease. **(B, C)** Axial PET/CT scans show increased uptake in the right lacrimal gland and in the right parotid gland, respectively (arrows).

Idiopathic orbital inflammation (IOI), also known as orbital pseudotumor, is a noninfectious, nonneoplastic inflammatory condition that can affect any orbital structure (32). It presents with acutely painful proptosis, eyelid edema, chemosis, and ocular motility restriction, sometimes mimicking infectious or neoplastic processes. On MRI, IOI appears as a poorly defined, enhancing mass that may involve the extraocular muscles (including their tendinous insertions), the lacrimal gland, or orbital fat, often with adjacent fat stranding. The affected tissues show T1 isointensity, variable T2 signal (often mild hyperintensity), and diffuse postcontrast enhancement. Unlike thyroid eye disease, IOI may show involvement of muscle tendons and usually presents with an absence of restricted diffusion, which helps differentiate it from most forms of lymphoma. Steroid responsiveness is another distinguishing clinical feature (13, 33, 34). Sclerosing orbital pseudotumor, historically classified under IOI, is now largely reclassified as a fibroinflammatory Immunoglobulin G4-related disease (IgG4-RD). IgG4-RD is characterized by lymphoplasmacytic infiltration rich in IgG4-positive plasma cells and storiform fibrosis (35). Specific staining of tissue for IgG4 should be requested, and elevation of IgG4 levels in serum is supportive of this diagnosis. IgG4-RD is a systemic disease and usually affects the lacrimal gland and can be associated with parotid lesions, retroperitoneal fibrosis, and pancreatitis. A characteristic finding on MRI is enlargement of the infraorbital nerves (**Figures 11**, **12**) (36, 37).

## Dermoid cysts

**Figure 13:**
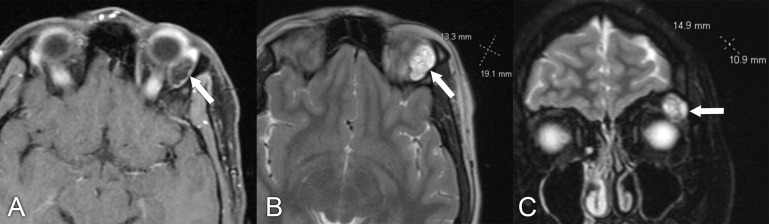
MRI findings in a 25-year-old woman who presented with a left supraorbital mass and fullness of the left upper eyelid. **(A)** Axial T1 postcontrast MRI with fat saturation shows a well-circumscribed lesion that measures 2.0 cm × 1.5 cm × 1.2 cm that contains suppressed fat and has rim enhancement (arrow). **(B)** Axial T2 MRI with fat saturation shows signal hyperintensity of the lesion (arrow). **(C)** Coronal T2 MRI with fat saturation image shows a heterogeneous signal (arrow). Overall, the lesion is radiographically consistent with a probable dermoid cyst. The lesion was excised with an intact capsule, and histology confirmed it to be a dermoid cyst.

Dermoid cysts are the most common benign lesions in children (38, 39), typically presenting as painless, noninflammatory masses often located near bony sutures (especially in the superotemporal orbit). On MRI, they appear as well-defined, nonenhancing lesions with high T1 signal intensity due to fat content and variable T2 signal, depending on their internal composition. Overall, these are benign, fat-containing lesions near bony sutures (**Figure 13**) (39).

## Orbital rhabdomyosarcoma

**Figure 14:**
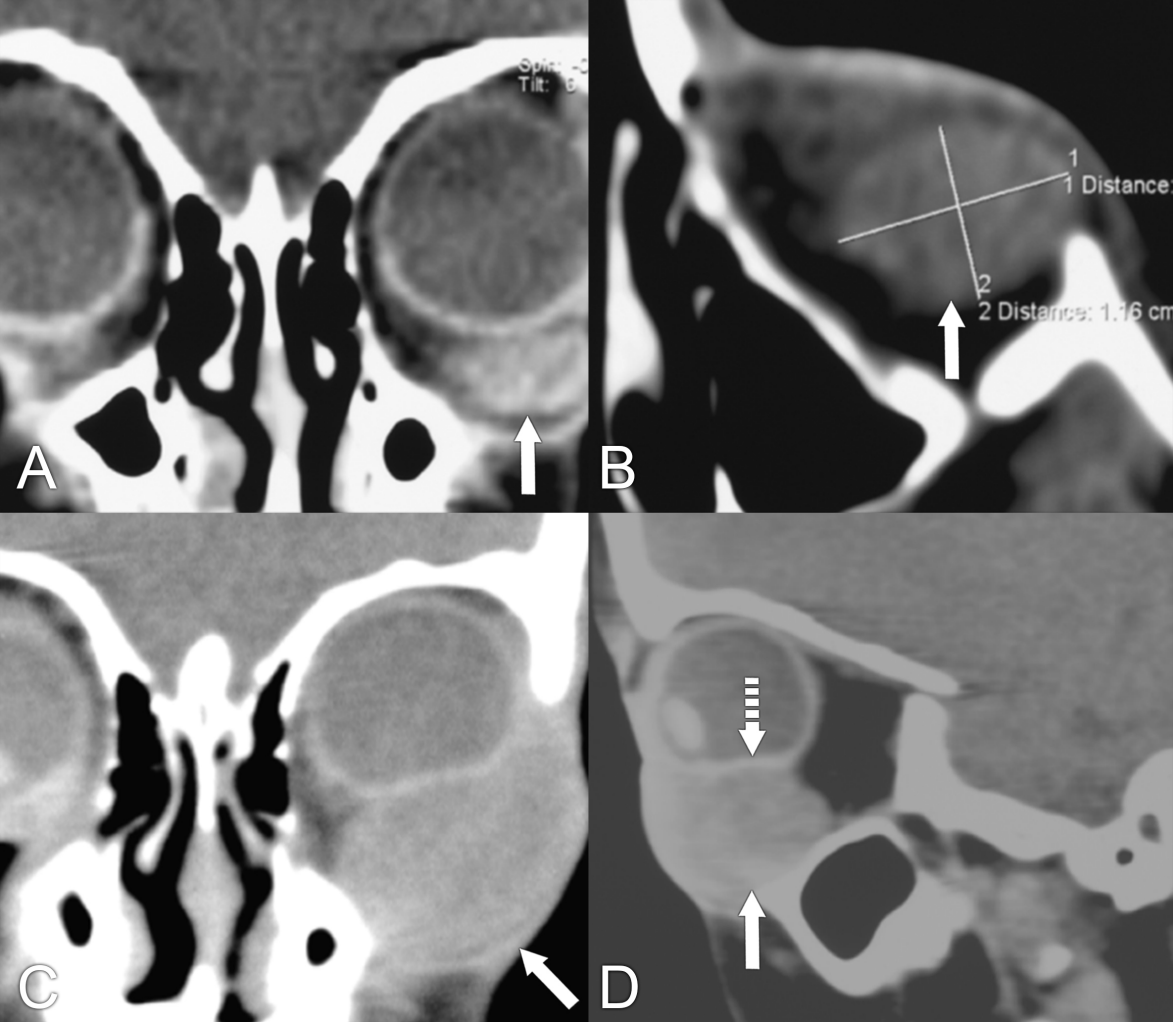
Imaging findings in a 4-year-old boy who presented with an acute onset of proptosis. **(A, B)** Coronal and sagittal CT with contrast, soft tissue windows acquired in the emergency department in his home country in the Middle East, show an inferior orbital mass (arrows). **(C, D)** Coronal and sagittal CT with contrast, soft-tissue windows acquired 4 weeks later, show significant enlargement of the mass, which is typical of rhabdomyosarcoma (solid arrows). The coronal image shows that the inferior orbital mass had almost tripled in size in just 4 weeks. The sagittal image shows globe distortion by the mass (dashed arrow). A biopsy of the orbital mass confirmed the diagnosis of embryonal rhabdomyosarcoma.

**Figure 15:**
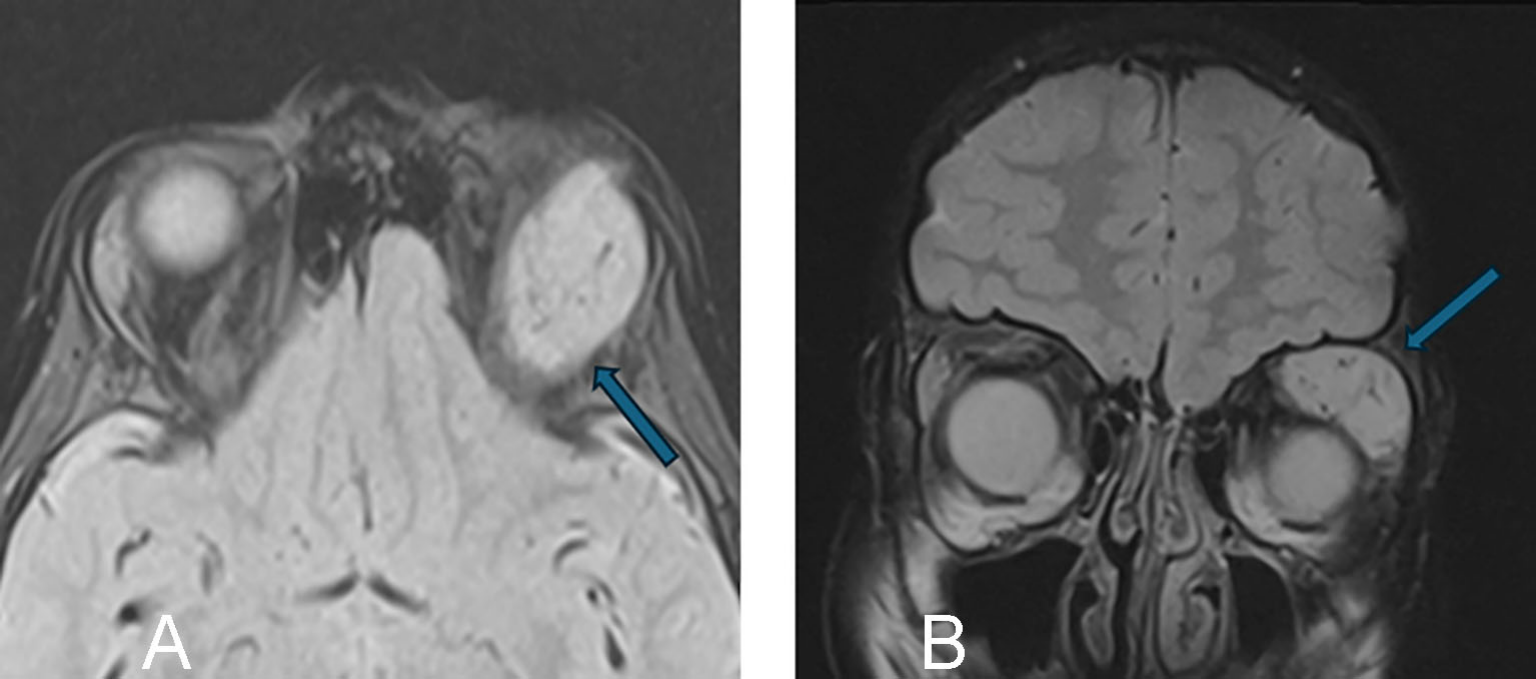
MRI images in an 8-year-old boy with embryonal RMS. MRI imaging is preferred, given the desire to avoid any radiation in children. However, CT scans are readily available and do not require sedation in young children. **(A, B)** Axial and coronal T2 MRI images show a large mass involving the left lacrimal gland (arrow).

Rhabdomyosarcoma (RMS) is the most common malignant mesenchymal tumor of childhood, with the orbit being the most frequent site in the head and neck region. RMS is the most common primary orbital malignancy in children and accounts for approximately 40% of orbital tumors in children, typically presenting between the ages of 5 and 10 years, with a slight male predilection (40, 41). The tumor usually arises from the extraconal compartment, most often in the superior orbit, and presents with rapidly progressive proptosis, ptosis, and sometimes clinical signs mimicking orbital inflammation, prompting urgent imaging. CT and MRI are both important in evaluating tumor extent, extraorbital or intracranial extension, bony erosion, and adjacent sinus involvement. The tumor is aggressive, and early diagnosis is essential. Although several benign and malignant orbital lesions may mimic RMS, the presence of unilateral, rapidly progressive proptosis in a child should raise a strong suspicion for this diagnosis (**Figure 14**). On MRI, RMS typically appears isointense to muscle on T1-weighted images, reflects variable signal intensity ranging from hypo- to hyperintense on T2-weighted images, and demonstrates marked, heterogeneous contrast enhancement (**Figure 15**). Orbital cellulitis is a key differential, as it can present similarly on imaging and clinically; however, the presence of fever, leukocytosis, and orbital fat stranding or abscess formation usually supports an infectious rather than neoplastic process (42).

## Optic nerve sheath meningiomas

**Figure 16:**
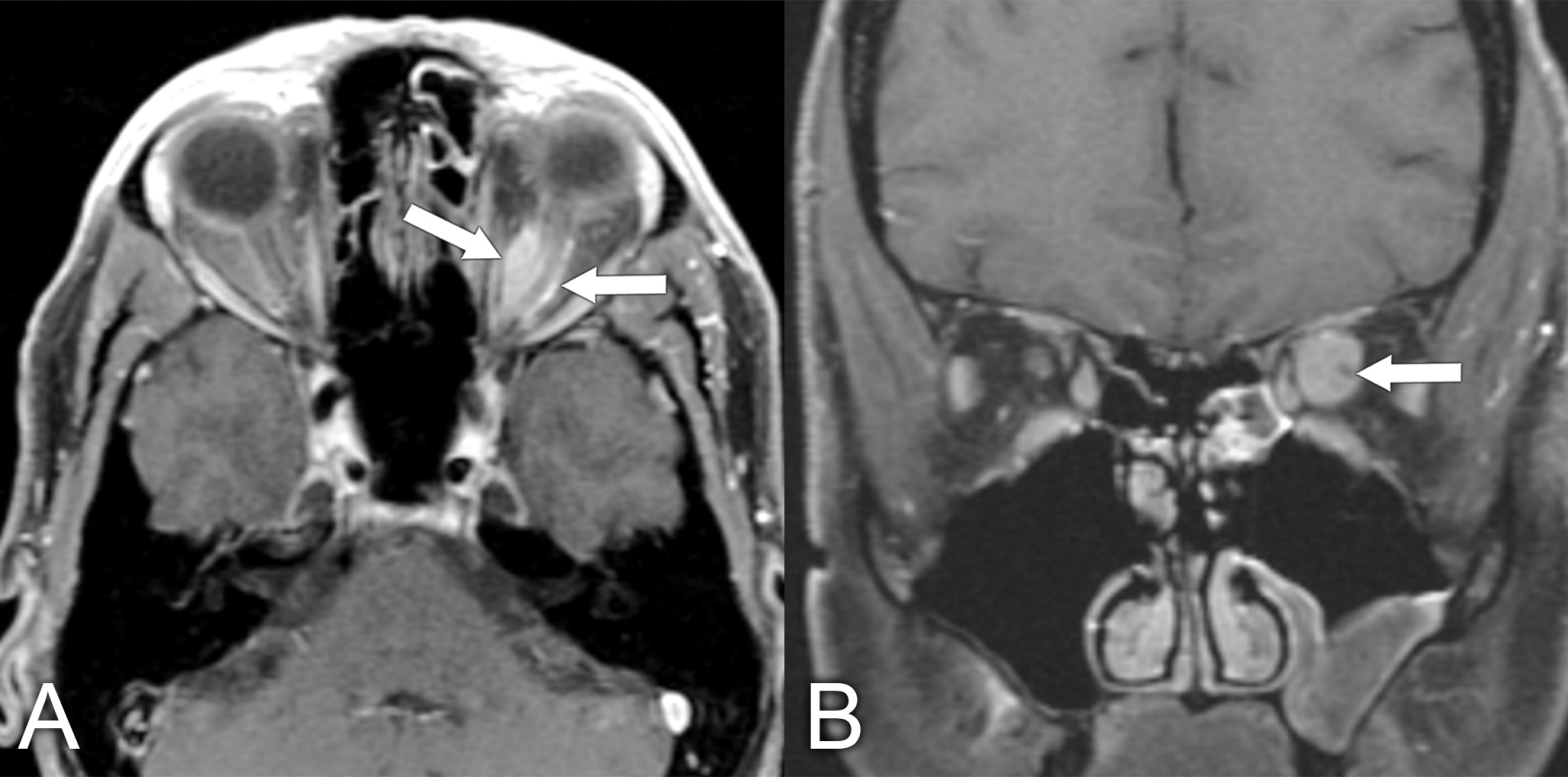
A 47-year-old woman presented with slowly progressive visual loss in the left eye. On exam, she had moderate visual loss with a relative afferent pupillary defect and a swollen left optic nerve head. **(A)** Axial T1 postcontrast MRI with fat saturation shows a lesion around the optic nerve with a “tram-track”-like appearance (arrows). **(B)** Coronal T1 postcontrast MRI with fat saturation in the same patient shows the enhancing mass surrounding the left optic nerve (arrow). The diagnosis, based on radiologic findings, was optic nerve sheath meningioma. The patient was treated with proton radiation therapy and experienced improvement in her visual function.

Optic nerve sheath meningiomas (ONSM) are rare, benign orbital neoplasms that arise from the meningothelial cells of the meninges surrounding the optic nerve. These lesions are usually unilateral in presentation and predominantly affect middle-aged women (fourth–fifth decade) (43). Bilateral cases can be seen in association with neurofibromatosis type 2 (44). The clinical course is usually insidious, with painless, progressive visual loss and atrophy of the optic nerve. Slowly progressive, mild proptosis can be seen in long-standing lesions. On MRI, segmental (intraorbital or intracanalicular) or diffuse nerve thickening can be seen. ONSM appears isointense on T1-weighted sequences and hypo- to isointense on T2-weighted sequences. Characteristically, there is intense homogenous contrast enhancement that gives the pathognomonic imaging feature of a “tram-track” appearance on axial images and a “doughnut” sign on coronal images (**Figure 16**), reflecting the thickening and enhancement of the lesion surrounding the normal, nonenhancing optic nerve (32).

## Orbital metastasis

**Figure 17:**
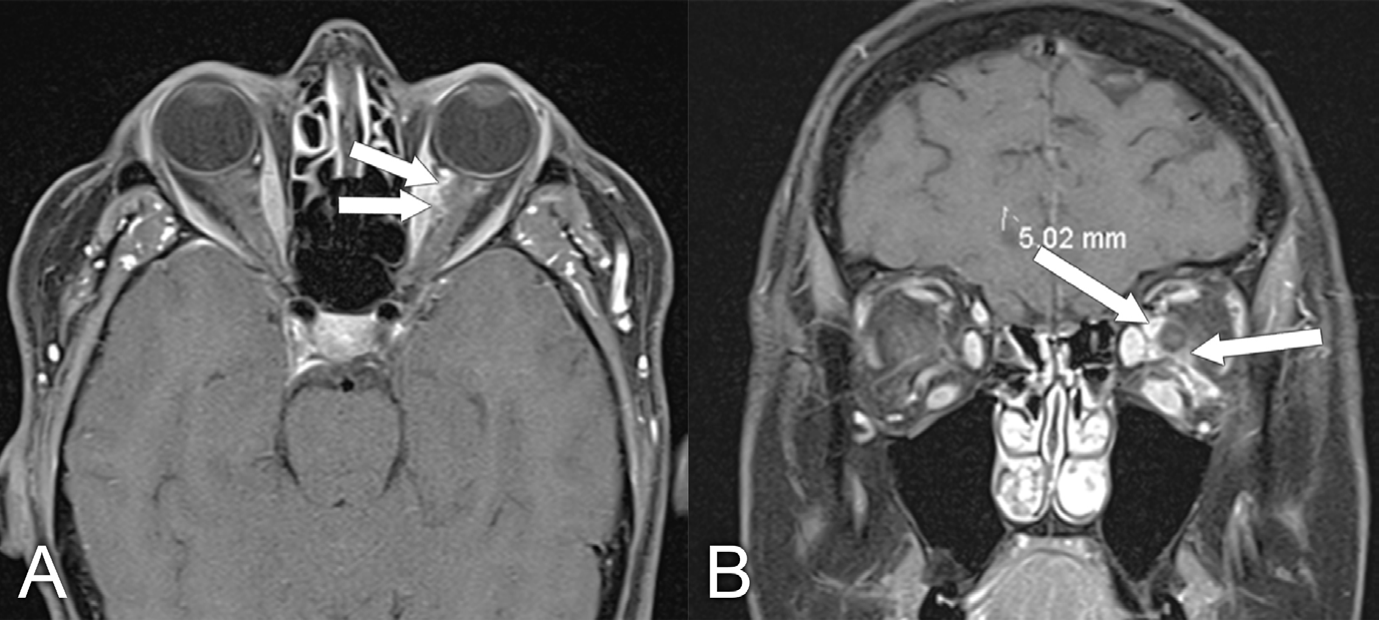
MRI findings in a 59-year-old woman with a history of metastatic breast cancer. For the past 3 months, she has noticed new-onset binocular diplopia. On clinical exam, she has mild left enophthalmos and restricted left eye movements. **(A)** Axial T1 postcontrast MRI with fat saturation shows enlargement of the left medial rectus muscle belly, as well as an intraconal infiltrative process in the left orbit (arrows). **(B)** Coronal T1 postcontrast MRI with fat saturation confirms enlargement of both the medial and inferior rectus muscles and an intraconal component of the lesion (arrows). This lesion was biopsied and confirmed to be metastatic breast carcinoma.

**Figure 18:**
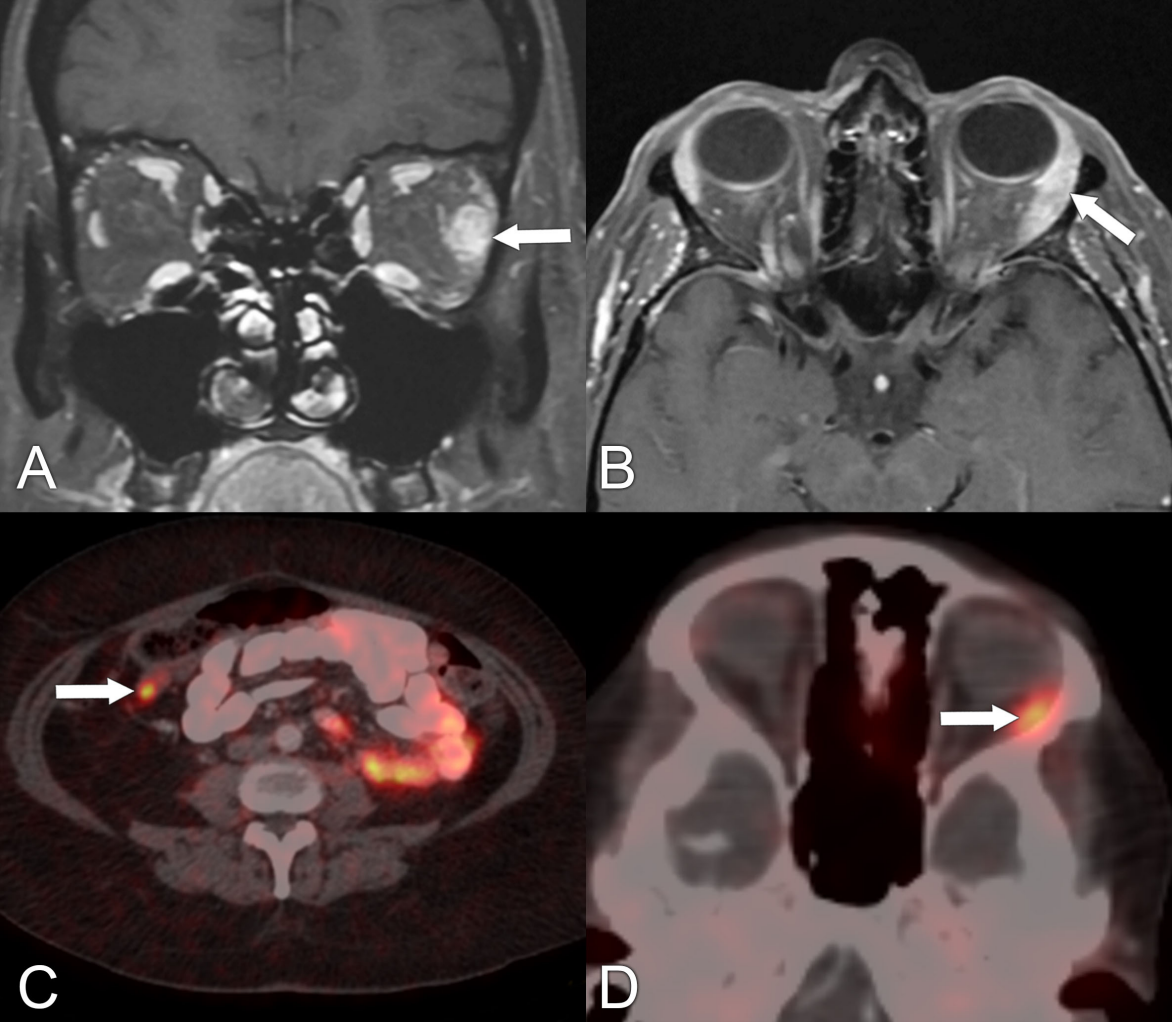
Magnetic resonance imaging and ^68^Ga-DOTATATE PET/CT in a 68-year-old woman with carcinoid tumor of the ileum with metastasis to the left orbit. **(A)** Coronal T1 postcontrast MRI with fat saturation shows a mass in the belly of the left lateral rectus muscle (arrow). **(B)** Axial T1 postcontrast MRI with fat saturation shows the lesion in the left lateral rectus muscle belly (arrow). **(C)** ^68^Ga-DOTATATE PET/CT shows a small but conspicuous focus of avidity localizing to the distal ileum (arrow). **(D)** ^68^Ga-DOTATATE PET/CT shows that avidity is also present within the lateral aspect of the left orbit along the lateral rectus muscle (arrow).

Orbital metastases from other sites account for 1%–3% of orbital tumors. The primary source of cancer may vary across regions; however, most studies show that metastatic breast cancer is the most common, accounting for 48%–53%, followed by metastatic prostate carcinoma, cutaneous melanoma, and lung cancer (45, 46). In children, neuroblastoma is the most common primary source (47). Orbital metastasis can involve the orbital bones and extraconal space, although intraconal involvement is also possible, and involvement of the extraocular muscles is often seen (**Figures 17**, **18**). The hallmark clinical presentation is painful proptosis, except in certain cases, such as scirrhous breast carcinoma metastases, where enophthalmos may occur due to infiltration and contraction of the extraocular muscles, particularly the rectus muscles. On imaging, both CT and MRI commonly reveal an enhancing, infiltrative mass, sometimes accompanied by bony destruction. DWI can aid in distinguishing orbital metastasis from lymphoma, as lymphomas typically show greater diffusion restriction. Additionally, PET-CT may incidentally detect orbital metastases and is useful for identifying the primary tumor and other metastatic sites throughout the body (48, 49).

In summary, orbital imaging is a critical part of the assessment and management of orbital tumors. Various modalities may be used depending on the clinical setting, age of patients, and other clinical considerations. Future directions may include the use of artificial intelligence (AI) and deep learning models in differentiating benign from malignant tumors, with the goal of decreasing interobserver variability and allowing for the extraction of quantitative imaging features that may not be appreciable by human observers (50).
